# O.5.2-4 ‘Experience of the menopause transition with reference to symptoms, health, and wellbeing, and how they impact motivators, facilitators, and barriers to physical activity engagement’

**DOI:** 10.1093/eurpub/ckad133.245

**Published:** 2023-09-11

**Authors:** Kelly Lee McNulty, Aoife Lane, Rosarie Kealy, Patricia Heavey

**Affiliations:** SHE (Sport, Health and Exercise) Research Group, Department of Sport & Health Sciences, Technological University of the Shannon, Athlone, Republic of Ireland; SHE (Sport, Health and Exercise) Research Group, Department of Sport & Health Sciences, Technological University of the Shannon, Athlone, Republic of Ireland; Waterford Sports Partnership, Waterford, Republic of Ireland; kelly.mcnulty@ait.ie; SHE (Sport, Health and Exercise) Research Group, Department of Sport & Health Sciences, Technological University of the Shannon, Athlone, Republic of Ireland

## Abstract

**Purpose:**

To explore experiences of the menopause transition (MT) of women living in Ireland with reference to symptoms, health, and wellbeing, and how they impact motivators, facilitators, and barriers to physical activity (PA) engagement.

**Methods:**

Eleven, perimenopausal, Irish women (age: 51 ± 3 years) participated in individual, online, semi-structured interviews. During each interview women were asked about their experience of the MT and its influence on PA engagement. All interviews were digitally recorded and transcribed *verbatim*, resulting in ≈ 51, 951 of words for descriptive and thematic analysis. Three to four themes were identified under the headings of motivators, facilitators, and barriers to PA engagement.

**Results:**

The MT was a significant event in most women’s lives that influenced PA engagement. The main motivators to engage in PA throughout the MT included safeguarding against long term health conditions, managing menopause symptoms, enjoyment through both socialising and achievement, as well as encouragement from family and role models. Many women discussed that feelings of community and collective experience through menopause fraternities, adapting and modifying, and medical supports were key factors that facilitated PA engagement throughout this life stage. There were a multitude of barriers that women in midlife faced before they could engage in PA, such as fears surrounding their capability to engage in PA, symptoms associated with the MT, the busyness of life and competing demands, as well as a lack of relatable opportunities and feelings of belonging within certain PA environments alongside other practicalities, including environment location and cost.

**Conclusion:**

The specific motivators, facilitators, and barriers to PA engagement that women experience throughout the MT are unique which is an important consideration for stakeholders when facilitating women to either continue or (re)introduce PA during this life stage.

**Support/funding source:**

Sport Ireland Research Grant Scheme.

